# A Method for the Symbolic Representation of Neurons

**DOI:** 10.3389/fnana.2018.00106

**Published:** 2018-12-18

**Authors:** Jose Juan Aliaga Maraver, Susana Mata, Ruth Benavides-Piccione, Javier DeFelipe, Luis Pastor

**Affiliations:** ^1^Departamento de Aeronaves y Vehículos Espaciales, Universidad Politécnica de Madrid (UPM), Madrid, Spain; ^2^Department of Computer Engineering, Universidad Rey Juan Carlos, Madrid, Spain; ^3^Center for Computational Simulation, Universidad Politécnica de Madrid, Madrid, Spain; ^4^Cajal Institute (CSIC), Madrid, Spain; ^5^Centro de Investigación Biomédica en Red sobre Enfermedades Neurodegenerativas, Madrid, Spain; ^6^Laboratorio Cajal de Circuitos Corticales, Centro de Tecnología Biomédica, Universidad Politécnica de Madrid, Madrid, Spain

**Keywords:** symbolic representations, schematic representation of neurons, neuron morphological and functional data visualization, pyramidal neurons, neuroscience information visualization, neuron functional data visualization

## Abstract

The field of neuroanatomy has progressed considerably in recent decades, thanks to the emergence of novel methods which provide new insights into the organization of the nervous system. These new methods have produced a wealth of data that needs to be analyzed, shifting the bottleneck from the acquisition to the analysis of data. In other disciplines, such as in many engineering areas, scientists and engineers are dealing with increasingly complex systems, using hierarchical decompositions, graphical models and simplified schematic diagrams for analysis and design processes. This approach makes it possible for users to simultaneously combine global system views and very detailed representations of specific areas of interest, by selecting appropriate representations for each of these views. In this way, users can concentrate on specific details while also maintaining a general system overview — a capability that is essential for understanding structure and function whenever complexity is an issue. Following this approach, this paper focuses on a graphical tool designed to help neuroanatomists to better understand and detect morphological characteristics of neuronal cells. The method presented here, based on a symbolic representation that can be tailored to enhance a particular range of features of a neuron or neuron set, has proven to be useful for highlighting particular geometries that may be hidden due to the complexity of the analysis tasks and the richness of neuronal morphologies. A software tool has been developed to generate graphical representations of neurons from 3D computer-aided reconstruction files.

## Introduction

It is essential to provide the scientific community with tools that make it easier for researchers to understand the acquired data and perform their associated analysis tasks faster and more effectively (Towns et al., 2012; Offerdahl et al., 2017).

In the particular case of the study of neuronal morphology, recent decades have seen continuous improvements in laboratory equipment and techniques. As a direct consequence, the size of the data sets that have become available has grown exponentially, up to a point where large collections of neurons are available (Ascoli et al., 2007; Staining, 2015). Researchers often must go through large numbers of cells, either to review, compare or characterize them — or to select samples for specific purposes such as cell models for setting up a computer simulation. Also, researchers often need to simplify operations such as analyzing diverse items, searching/recovering data similar to a particular one used as a template, etc. Clearly, any help for tasks such as discovering trends or singularities or establishing theories would be very welcome (Morales et al., 2011; Al-Awami et al., 2014; Toharia et al., 2016). The availability of a common representation system would certainly facilitate performing these operations in an unambiguous way, while also highlighting their most relevant features for each specific study.

Regarding the representation of neurons, currently available commercial software (Glaser and Glaser, 1990; Bitplane, 2012; MBF Bioscience, 2018) facilitates the generation of detailed representations of 3D neurons that can then be used for anatomical studies. These digital representations allow the automatic computation of quantitative measures such as dendritic length, number of nodes (points where the dendritic tree divides into two branches), number of terminations, etc. Although these numerical values can characterize neurons according to a pre-specified set of variables, there are certain aspects that are not easily captured by them, such as arborization patterns, spatial relationships, element distribution, etc. Approaching this from a different angle, strategies for data visualization provide a useful resource for the analysis and exploration of complex data (Lempert, 2002; Unwin and Chun-houh, 2008; Bankman, 2009; Ward, 2015); consequently, several techniques have been proposed for the visualization of detailed neuronal morphologies (Lasserre et al., 2012; Brito et al., 2013; Garcia-Cantero et al., 2017). However, even though visualizing cells helps researchers acquire an overall idea of each neuron’s morphology, the 3D structure of these neuronal representations means that the final images are highly dependent on the subjective interpretation of the user, who will chose the direction of projection. Neurons have an intricate geometry, and 3D visualizations commonly present perspective artifacts. These two characteristics make it difficult to discern geometrical details and to distinguish certain morphological features. Again, the availability of accessible representations would simplify the analysis task, especially in the case of large datasets.

This paper presents a novel representation framework for the visualization of neurons from anatomical 3D reconstructions, facilitating the visual exploration and analysis of cells. Specifically, the proposed approach presents the following advantages:

• It provides unambiguous 2D neuron representations, eliminating the occlusions that occur in Z projection images, while still capturing the whole 3D anatomy of the cell.
• It defines visual abstractions for neurons, based on circular dendrograms, where the selected morphological features are depicted in a symbolic way. This allows the emphasis of the most significant information for a specific task, placing less importance on less relevant features (or even hiding them completely).
• It can be interactively parameterized in order to represent different variables, thereby also facilitating sorting and filtering operations according to different characteristics.

The proposed representation methodology provides a new resource for communicating information or performing visual analysis of morphological features for large populations of neurons. Although this method has been conceived specifically for the description of individual neuron morphologies, it can be integrated into more powerful combined morphological and physiological representations within a multiresolution framework. This tool is publicly available at https://piziadas.com/hugo.

The following sections include a brief description of the state of the art, a summary of the design principles and main features of the proposed method, as well as an analysis of how this method can help to perform different analysis tasks. Finally, the main conclusions are presented, along with proposals for future work.

## State of the Art

There are many ways to define a representation; according to Marr and Nishihara (1978), “a representation is a formal system for making explicit certain entities or types of information, together with a specification of how the system does this”. Other definitions stress the idea that representations provide images or concepts that help us to build a mental model of any object. Representation systems, therefore, have been used for two main purposes: providing means for transmitting information, on one hand, and for systematizing ideas and facilitating the development of mental visualizations of objects or concepts, on the other. Both of these have been fundamental contributors toward human progress, especially for the development of science and technology (Evagorou et al., 2015).

**FIGURE 1:**
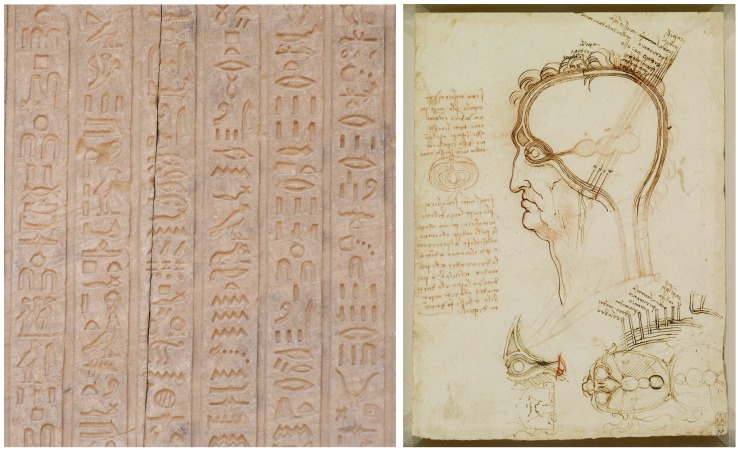
Examples of graphical representations from different historical periods. **(Left)** Hieroglyph inscribed on one of the walls of the temple of Dendara (Egypt), dedicated to the goddess Hathor. **(Right)** Leonardo da Vinci, Quaderni d’anatomia (1490): the central nervous system and cranial nerves. The main drawing shows the layers covering the brain compared to the layers of an onion cut in half (on the left of the image). At the bottom of the drawing, the ventricles viewed from above are illustrated, including the optic and auditory nerves entering the anterior ventricle.

Historically, humans have been developing graphical representations for many different purposes for at least 73,000 years (Callaway, 2012; Pike et al., 2012; Henshilwood et al., 2018; Hoffmann et al., 2018), first in caves and on rocks in a wide diversity of geographical areas. Later, virtually every civilization produced different artistic or religious paintings and engravings as distinctive cultural features. More related to the subject of this paper, drawings have also been customarily used in many branches of science, such as medicine and biology, where detailed illustrations have been widely used to document new findings. Figure 1 presents some examples of representations —from very different time periods— which have been used for different purposes, and include symbolic and realistic representations, as well as actual images or pictograms. Each representation conveys some information in its own particular way.

The case of schematic representations in computer architecture is very relevant to design or representation methods in neuroscience, given the similarities between the two cases regarding issues such as system complexity and the need to provide users with simultaneous access to fine details, long-range interaction and system-level information. The solutions devised in computer architecture were based on defining hierarchies of description levels associated to synthetic representations for each level; Tanenbaum and Goodman (1998) structure a computer as a series of abstractions, each one built upon the ones below it. The lowest level is the digital logic level and it deals with gates that are usually represented by symbols that hide their internal structure. The highest level consist of the so-called high-level languages used by application programmers to solve specific problems. This allowed engineers to focus on specific features at the most appropriate level of detail for each task, working only with the information that was relevant at any given moment. The availability of these abstraction levels and their associated representations were essential for the purpose of designing, developing and analyzing modern high-performance computers.

**FIGURE 2:**
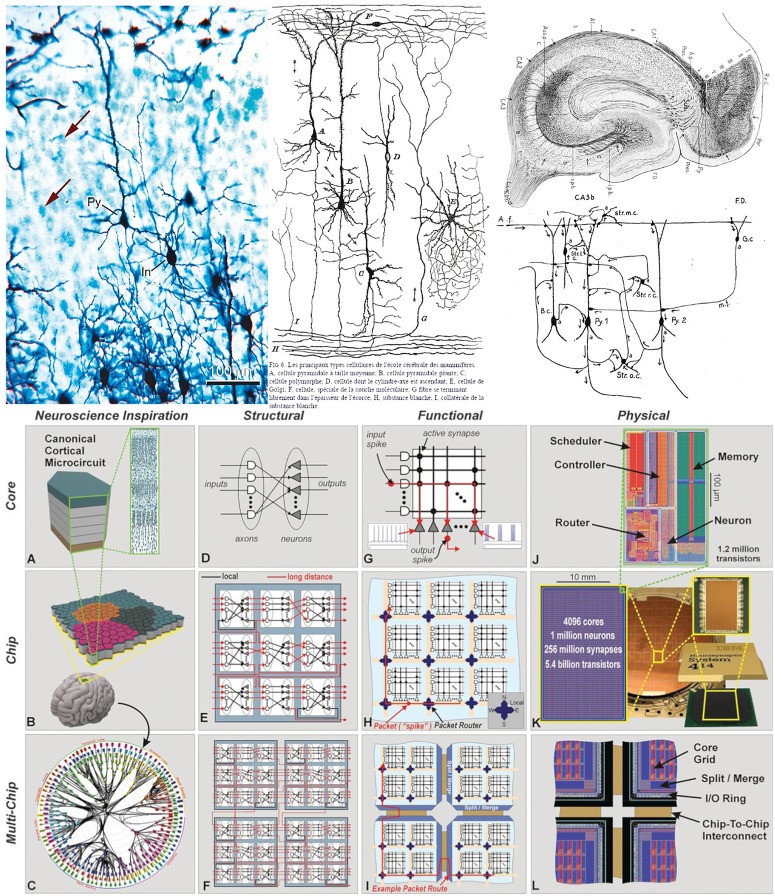
**(Upper)** Left column: preparation of mouse cerebral cortex stained with the Golgi method and counterstained with the method of Nissl. With the method of Golgi, only the cell body and its processes are stained in black, whereas with the method of Nissl, only the cell bodies are stained in blue (arrows indicate some stained cells). Note that with the Golgi method, only a small proportion of the cells are stained. In, interneuron; Py, pyramidal cell. Scale bar: 100 mm. Middle and right column: theoretical direction of transmission of impulses in cortical circuits published by Cajal in 1894 (middle column) and Lorente de Nó in 1934 (right column). The diagrams stem from the neuron doctrine and the law of dynamic polarization of nerve cells (and are based on sparse anatomical data). Taken from DeFelipe (2014). **(Lower)** Networks inspired by neural architecture (TrueNorth architecture). **(A)** Neurosynaptic core inspired by a canonical cortical microcircuit. **(B)** Network of neurosynaptic cores inspired by the cortex’s two-dimensional sheet. **(C)** Multichip network inspired by the long-range connections between cortical regions. **(D)** Structure of a neurosynaptic core. **(E,F)** Multicore networks. **(G)** Functional view of core. **(H)** Functional chip architecture. **(I)** Routing network across chip boundaries. **(J)** Physical layout of core in 28-nm CMOS. **(K)** Chip layout of 64-by-64 core array, wafer, and chip package. **(L)** Chip periphery to support multichip networks. Taken from Merolla et al. (2014).

In the field of neuroscience, realistic drawings and images have been the most common way of documenting scientific discoveries. For example, Cajal’s beautiful drawings and diagrams of single cells or simple circuits are well known (DeFelipe and Jones, 2013; DeFelipe, 2014), and were essential for the documentation and dissemination of his findings. Indeed, many of the illustrations by Cajal and other scientists at that time were composite drawings that synthetically showed the complex texture of a given region of the nervous system. This is because, for example, using the method of Golgi (the most common method at the time), they could visualize relatively few cells in a given histological preparation [this can be seen when a section is stained with this method and counterstained with the method of Nissl (Figure 2, upper panel, left column)]. Thus, as dealt with in DeFelipe (2017), in order to generate a circuit diagram as shown in the middle column of the upper panel of Figure 2, Cajal had to interpret several microscopic preparations and highlight the key features of the structure being studied. In other words, these circuit diagrams were produced from interpretative skills based on sparse neuroanatomical data. As discussed in DeFelipe (2017), Cajal proposed that, in general, neurons could be divided into three functionally distinct regions: a receptor apparatus (dendrites and soma), an emission apparatus (axon) and a distribution apparatus (terminal axonal arborization), and the early neuroanatomists used arrows in their diagrams to indicate the direction of the nervous currents. Thanks to the theory of dynamic polarization, it was possible for Cajal and others to trace and interpret the flow of information in complex microcircuits of the nervous system. For example, this is how his pupil Lorente de Nó described the complex connectivity of CA3 in 1934 (Figure 2, upper panel, right column):

Arrows indicate the direction of transmission of the impulses according to Cajal’s law of axonal polarization. If this law is not accomplished […] the interpretation of the diagram would be quite different.

With the improvement of microscopy and physiological techniques, the complexity of the acquired data has been steadily increasing. In this context, non-realistic representations have been used for a variety of purposes, such as describing individual features of single cells and circuits as well as functionality. For example, networks inspired by neural architectures helped to develop silicon technology (Figure 2, bottom panel). In these cases, there is a trend toward using simplified, schematic representations to enhance the specific aspects that are most relevant for dissemination depending on the particular research findings in question. Still, many of the representations used are only capable of providing partial views of different aspects, and have not been conceived as tools to facilitate systematic analysis operations.

Regarding this point, the main goal of the techniques proposed in this paper is the design of a representation system for neurons that provides unambiguous 2D representations of their 3D structure. This method has been primarily designed to facilitate the visual analysis of neuron morphology, as well as to evaluate how different sets of computed features vary in terms of their constitutive elements.

## Materials and Methods

The first step of the method presented here is to depict the 3D cells’ structure using a 2D representation that encodes the morphology of the dendritic and axonal trees unambiguously. This structure will then act as the underlying canvas over which other additional information will be mapped, whenever that information is relevant for a specific task. It should be noted that, in this work, only morphological features have been used. Nevertheless, the methods presented here can be easily applied to the mapping of functional/molecular information or features.

Four key issues have guided the design of the representation system proposed here:

• Which neuronal elements and characteristics are represented
• Which visual resources are used for neuronal representation
• How this information is represented
• How users interact with the data and representations

The way in which these issues are dealt with is necessarily dependent on user goals and expectations.

Regarding neuronal elements and characteristics, the representations proposed here include diameters; lengths; number of endings and nodes; surface areas; volumes; and angles between branches.

The set of graphical resources to be used within these representations has been selected to be easily interpretable, facilitating the automatic mapping of information from the computed features onto the representations. For this process, the system is initially based on 3D computer-aided reconstruction files stored following Neurolucida file format (.dat, .asc) or neuromorpho file format (.swc). Visual characteristics such as color, size and line width can then be used to represent the morphological characteristics, and are easily computed from the cell descriptions, yielding a straightforward mapping between variables and visual representations. Finally, the spatial and geometrical distribution of some of the representation elements can also be modified according to the values of some pre-specified features. How this information is represented and how users interact with the data will be described in detail in the following sections.

The input data used in this work consist of 3D reconstructed apical and basal arbors from 17 human and mouse pyramidal neurons (5 and 12, respectively) that were intracellularly injected with Lucifer Yellow (LY). Human tissue samples were obtained from layer III of the human cingulate and temporal cortex and the CA1 region of the hippocampal formation (see Garey, 1994) of 3 human cases obtained at autopsy (2–3 h post-mortem; 2 males aged 40 and 45, 1 female aged 53; kindly supplied by I. Ferrer, Instituto de Neuropatología Servicio de Anatomía Patológica, IDIBELL-Hospital Universitario de Bellvitge, Barcelona, Spain and Dr. Ricardo Insausti, UCLM, Albacete, Spain).

Mouse tissue samples were obtained from the primary somatosensory cortex [layers II–VI; (Franklin and Paxinos, 2004)] of C57BL/6 adult (8-week-old) male mice that were overdosed by intraperitoneal injection of sodium pentobarbitone and perfused intracardially with 4% paraformaldehyde. Vibratome sections (300 μm for human tissue and 200 μm for mouse tissue) were obtained in the coronal plane. Sections were then labeled with 4,6 diamino-2-phenylindole (DAPI; Sigma, St. Louis, MO, United States) to identify cell bodies. Pyramidal cells were then individually injected with LY (8% in 0.1 M Tris buffer, pH 7.4) in the corresponding brain regions described above. LY was applied to each injected cell by continuous current until the distal tips of each cell fluoresced brightly, indicating that the dendrites were completely filled and ensuring that the fluorescence did not diminish at a distance from the soma. Following the intracellular injection of pyramidal neurons sections were immunostained for LY using rabbit antisera against LY (1:400 000; generated at the Cajal Institute) diluted in stock solution (2% bovine serum albumin, 1% Triton X-100, and 5% sucrose in PB). The sections were then incubated in biotinylated donkey anti-rabbit IgG (1:100; Amersham, Buckinghamshire, United Kingdom) and Alexa fluor 488 streptavidin-conjugated (1:1000; Molecular Probes, Eugene, OR, United States). Finally, sections were mounted in 50% glycerol in PB. Further information regarding tissue preparation, injection methodology and immunohistochemistry processing is outlined in Benavides-Piccione et al. (2013). The injected cells were fully imaged at high magnification using tile scan mode in a Leica TCS 4D confocal scanning laser attached to a Leitz DMIRB fluorescence microscope. Fluorescent labeling profiles were imaged, using an excitation wavelength of 491 nm to visualize Alexa fluor 488. Consecutive stacks of images at high magnification (×63) were acquired to capture dendrites along the apical and basal dendritic arbors. Neuron morphologies were extracted in 3D using Neurolucida Confocal package (MicroBrightField). We used additional digital reconstructions that were obtained from Neuromorpho.org (Ascoli et al., 2007).

## Circular Dendrograms for Neuronal Description

**FIGURE 3:**
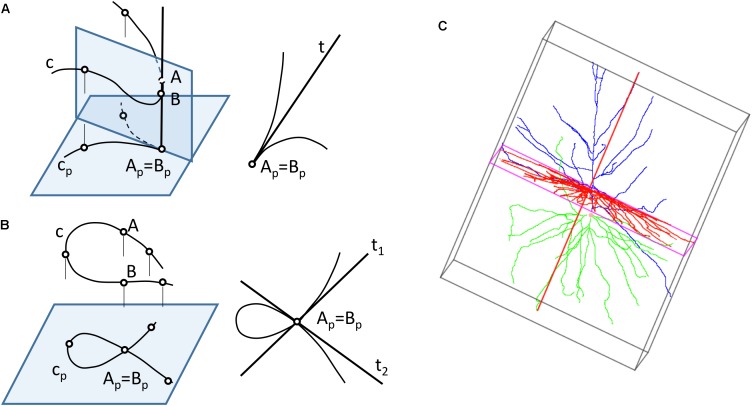
Computing 2D projections of neurons. **(A)** The projection of a space curve may result in curves with singularity points, such as cusps **(B)** If two points of the curve are aligned along a projection line, the projected curve will have multiple points. **(C)** A bounding box can be generated around a neuron to find the direction of projection that provides a projection with maximum area.

Neurons can be represented in 2D by projecting their 3D shape onto planes — a process that preserves more or less geometric details depending on which projection plane has been chosen. However, 2D projections present some problems: first, moving from *R*^3^ to *R*^2^ leads to loss of information; recovering the original 3D shape requires at least a second projection over a different plane. Also, projections from 3D to 2D can produce additional distortions (Figure 3), since the projections of the points that lay in the same projection ray overlap in the 2D plane, giving rise to topological singularities that can greatly modify the appearance of the cell (Sharma, 2010). Lastly, in order to compute a projection, it is necessary to select which plane will give the best projection for the actual data and operator goals. This is strongly related to the data acquisition process, which is commonly achieved by starting with a stack of microscopy images and producing a series of tracing points located in a set of parallel planes (Z coordinates can be systematically grouped).

The most common projection used for computing the neuronal representations is a cylindrical orthogonal projection in the XY plane — a process that can generate singularity points (Figures 3A,B).

The projection direction for these cylindrical projections is usually selected by the operator, although whether this is the case or not will very much depend on his or her experience, and also on whether the projection is aimed at acquiring the whole neuronal structure or only a part of it. In any case, this step can be further optimized by selecting the projection plane that captures the greatest area of the cell. Bounding box computation (O’Rourke, 1985; Swart, 1985; Barber et al., 1996; Avis et al., 1997; Verleysen and Vleeschouwer, 2016) can facilitate this process (Figure 3C).

The morphology of a neuron can be schematically represented by a graph that shows the morphological tracing points corresponding to neurite bifurcations, connected following the digitized dendritic and axonal trajectories. Assuming, without loss of generality, that branches always bifurcate into just two sub-branches, dendrites and axons can be represented by binary unbalanced trees. Geometric detail can be easily reduced by eliminating neurite curvature, replacing real trajectories with the line segments that result from straightening each of the neurite fragments comprised between subsequent bifurcations, or between a bifurcation and either the soma or its ending point (Van Pelt et al., 2001).

**FIGURE 4:**
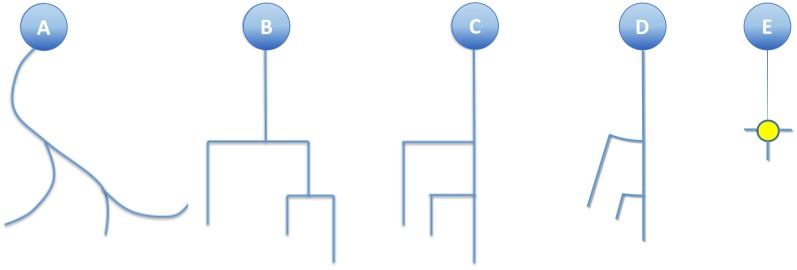
**(A)** A real neurite fragment. **(B)** A classical dendrogram representation of the same neurite. **(C)** The binary tree in B has been modified by laterally displacing each of the secondary branches. Here the neurite bifurcation nodes can be identified as the segments orthogonal to the neurite main axis. The circular dendrogram presented in **(D)** is obtained by repositioning the branches radially where bifurcation nodes are represented by circular arcs. **(E)** This model can still be simplified by substituting its branching pattern with a symbol that encodes a specific morphological feature (in this case, the number of branch endings).

This simplification, combined with the substitution of bifurcation points with small orthogonal segments, gives rise to the classical dendrogram representation of neurons as binary trees (Figure 4B). This abstraction is helpful for constructing a conceptual scheme for the overall cell structure, facilitating the perception of aspects such as level of subdivision or branching order. However, neurons with a large number of neurites or a high branch order result in huge trees with a complex structure covering large areas within the representation space. This drawback can be alleviated by aligning the main branches and displacing all the secondary branches laterally such that they point in the same direction (Figure 4C). This modification produces clearer, crisper representations that require less representation space. This spatial arrangement can be further optimized by distributing the branches following a circular pattern (Figure 4D). This modification gives rise to the so called circular dendrograms, which provide 2D representations of neuronal morphology that can be unambiguously interpreted, and are also independent from the operator that generated the representations or the projection plane/direction. Additionally, circular dendrograms provide representations that are closer to real neuronal morphologies, facilitating their interpretation. Lastly, this model uses the available space optimally, assigning larger areas to distal neurite segments, which present larger numbers of branches, as is the case in real neurons, where distal branches typically spread over larger regions.

Regarding dendrograms, it should be noted that losing the actual curved trajectories for the different neurite fragments is not disadvantageous for many tasks. Indeed, simplifying neurite shape provides a schematic, abstract view of the cell morphology that is much easier for a human observer to interpret. In any case, combining circular dendrograms with real 2D projections or 3D representations provides users with complete and clear information.

**FIGURE 5:**
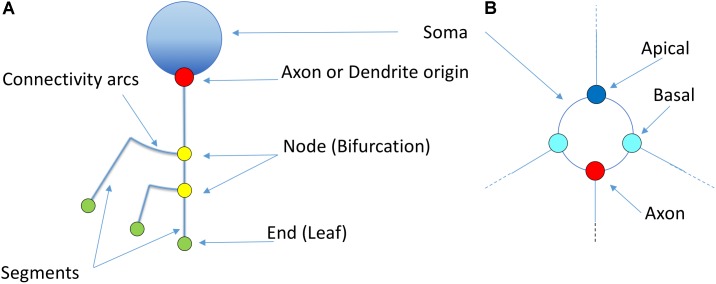
Basic elements of the proposed representation model **(A)**. The soma is idealized as a circumference from which dendrites and axons originate. The starting points of the different neurites can be highlighted with a dot (Red: axon; Blue: apical; Cyan: basal). Straight segments connect two bifurcation nodes (yellow) or a bifurcation node and an ending point (green). An arc is placed at each bifurcation point in order to connect the main branch with the secondary one. **(B)** Shows a detailed view of the first order dendrites and axon, representing two basal dendrites (cyan), one apical dendrite (dark blue) and the axon (red). Proximal nodes help identify the number of first order branches.

Figure 5 illustrates other basic elements of this representation model, such as the symbols used for representing somas, nodes, etc. The starting points of different neurites can be color-coded to facilitate visual analysis tasks.

**FIGURE 6:**
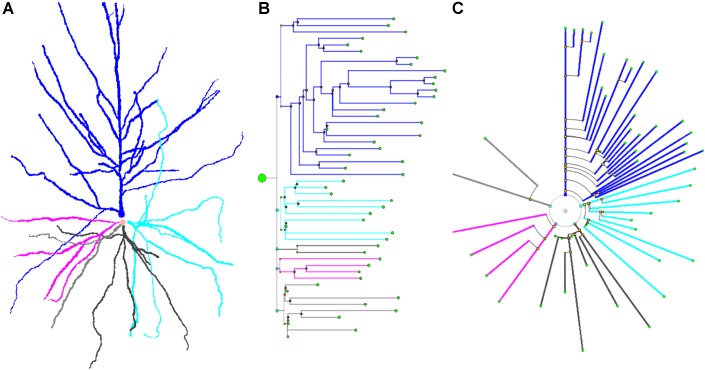
A human cortical pyramidal neuron and two dendrogram representations. **(A)** presents a standard 2D projection of the real neuronal morphology, while **(B)** and **(C)** show two different dendrograms for the same cell (**B** presents a classical dendrogram and **C** the proposed circular dendrogram). Corresponding neurites on each representation are coded with the same color to facilitate their identification in the different representations.

Figure 6 shows a real pyramidal neuron, its representation using a classical dendrogram, and the proposed circular dendrogram. The radial distribution in circular dendrograms results in a compact depiction, which resembles the original orientation of the main dendrites and makes it easier for users to mentally visualize the match between real and dendrogram neurites.

The 2D circular dendrograms presented in Figures 5, 6 are the basis of the representation system proposed in this paper. They provide a visual abstraction of neuronal anatomy which is obtained via an evolution of the widely known binary tree representation. It should be noted that these circular dendrograms can be automatically computed from raw morphological tracings, presenting details in a way that facilitates human understanding of different features. Furthermore, the visual characteristics of these diagrams and the spatial distribution of their elements can be modified in order to encode different data, as will be explored in the following sections. Please note that radial distributions have often been used in the field of information visualization (Draper et al., 2009).

## Circular Dendrograms for Neuronal Morphological Analysis

Symbolic representations can be designed and modified to meet precise requirements, such as those needed for a particular analysis job. This kind of representation can be customized for almost any purpose; designers have quite a large degree of freedom to decide which aspects of the representation will be over- or underemphasized, since they are freed from the need to accurately display the actual features from the data being represented. Consequently, displaying the structure of neurons in a schematic, easy-to-grasp way is not the only advantage offered by circular dendrograms; each basic diagram can be modified to emphasize those aspects which are most relevant, in order to facilitate the visual analysis operations related to a specific task. Also, different variables can be mapped onto these diagrams using different visual resources. In this way, this kind of schematic representation can be adapted to present additional information at the request of the user.

The circular dendrograms presented here have been developed within an exploratory analysis framework, conceived with the analysis of neuronal morphology in mind, although this framework has already also been extended to deal with functional data (Galindo et al., 2016; Toharia et al., 2016; NeuroLOTs, 2018). In this framework, operators can use a number of tools to carry out procedures such as cluster analysis, search by content, analysis of the spatial variation of specific features, etc. Several other operations are performed routinely, such as cell or neurite selection and filtering, navigation through populations of acquired neurons or within neuronal morphologies.

Circular dendrograms are useful in this context, providing views that can highlight different features depending on their relevance for a particular purpose. This section presents a number of representations that can facilitate the perception of certain cell features through the variation of aspects such as segment length; angular arrangements; and line width and color.

### Segment Length in Circular Dendrograms

Segment length in circular dendrograms can be made proportional to real neurite segment lengths; we call this representation mode Real Length Mode (RLM). This facilitates, for instance, performing detailed studies of morphological aspects related to the actual size of the neurites. In addition, using real lengths in the representations allows the mapping of other features over the neurites (for example, the actual location or density of dendritic spines), which gives users an accurate impression of the variation of these features across dendritic and axonal trees (see section “Other Resources”).

The length of the segments in the dendrogram can also represent the radial distance from node to soma. This can be used to perform Sholl’s analysis (Sholl, 1953). We call this representation mode Radial Mode (RM). Also, in a fourth mode, called Length Mapping Mode (LMM), the length of each segment of the representation can represent a different variable — for instance, mean diameter of the segment, side-area, etc.

**FIGURE 7:**
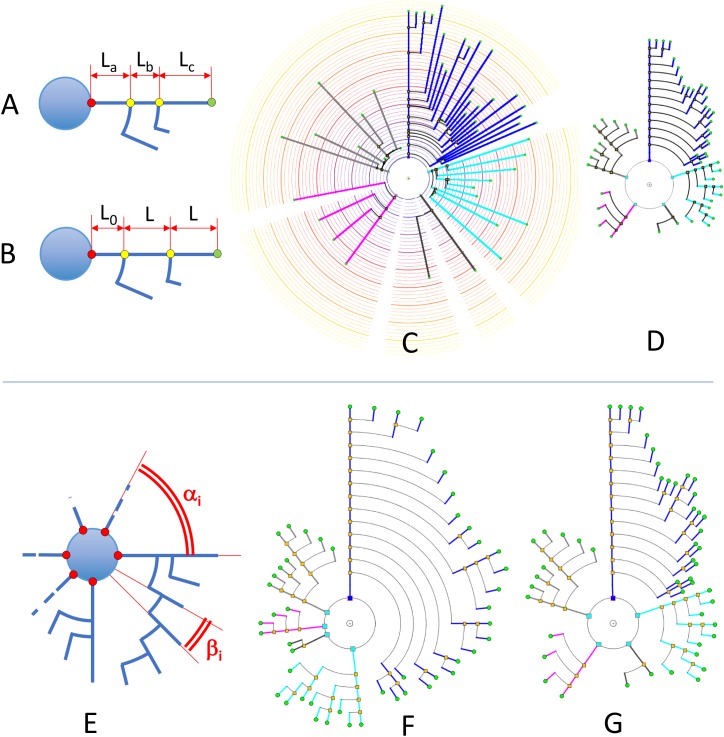
*Upper*: In RLM and RM, segment lengths in circular dendrograms represent real neurite lengths as measured in morphological tracings or radial node to soma distances **(A,C)**. In UM, using fixed segment length in dendrograms facilitates other operations where neurite or branch length is irrelevant, such as for the analysis of arborization patterns **(B,D)**. In UM, L_0_ is an arbitrary distance to facilitate the visual interpretation of the model. *Lower*: diagram **(E)** shows the two different angles that can be modified in circular dendrograms. The first of them, α, is the angle assigned to each dendritic or axonal arbor. The second one, β, is the angle assigned to each segment or terminal branch at each bifurcation node. In **(F)**, the dendrogram has been arranged with ENDM (constant angular distribution of neurite terminals) by using a variable angle α, and keeping β constant. **(G)** On the other hand, has been obtained with NDM (constant angular distribution of neurites). In this case, α is constant, with different β angles per neurite arbor.

Segment length can also be given less prominence whenever other features are being considered and segment length is not relevant. For example, using equal lengths between nodes can allow users to better perceive neurite arborization patterns. We call this representation mode Unitary Model (UM). Figures 7A–D shows a comparison between some of these options for a real pyramidal cell. The number of dendrites, the complexity of their arborization patterns and the number of branch levels and endings are easier to grasp when uniform lengths are used.

### Angular Arrangements

Users can modify the way the different dendrogram elements are angularly arranged within the diagram. This distribution is governed by four degrees of freedom that define the final representation:

• Angular sector covered by each primary branch
• Angular sector covered by each individual neurite ending
• Ordering criteria for the different neurites
• Ordering criteria for the different neurite branches and branch endings

These options will be studied here, grouped into two cases: angular sectors and ordering criteria.

#### Angular Sector Distribution

The width of the circular sector assigned to each neurite or neurite branch can be adjusted. This allows the emphasizing or de-emphasizing of neurites (or the information they convey) within the whole dendrogram. In the End-Node Distribution Mode (ENDM), the angular distance between each terminal node of the neuron is kept constant. In the Neurite Distribution Mode (NDM), the angular sector allocated to each neurite originating from the soma is constant. The terminal nodes of each main neurite are distributed by following an ENDM within that angular sector. Assigning uniform circular sector angles to neurites in dendrograms —as in NDM— facilitates user perception of the total number of neurites within a cell as well as its high-level neurite structure.

Additionally, in the Weighted Distribution Mode (WDM), different correction weights, *C*_ij_, can be applied to each neurite *i* and branch ending *j*, resulting in non-uniform angular distributions. This option allows additional space to be allocated to certain neurites or branches whenever users wish to devote additional representation resources to any neurite or branch in order to highlight specific features.

With respect to angular sector size (Figure 7E), there are two families of angles that have to be considered:

• α_i_: Aperture angle of the circular sector assigned to the *i*_th_ neurite arbor.
• β_i_: Aperture angle of the circular sector between two segments at a bifurcation node within the *i*_th_ arbor (equivalent to the angle assigned to each terminal branch, and ending, within the *i*_th_ arbor).

Let *E* be the number of terminal nodes (branch endings), and let *N* be the number of trees (neurites) to be represented in the dendrogram. Then, the two sets of angles α_i_ and β_i_ can be computed in different ways:

• Assigning constant angles, β_i_, per branch ending: the constant value of β assigned to each ending can be computed as:
  $$\beta =K=\frac{360}{E}$$

In this case, the angle spanned by each neurite arbor will be α_i_ = *E*_i_^∗^β

• Assigning constant angles, α_i_, per neurite arbor: each constant angle occupies a circular sector of uniform width α:
  $$\alpha =\frac{360}{N}$$

Now, each neurite will have to pack its segments, nodes and branch endings more or less tightly depending on the number of branch endings per arbor. The angle between two consecutive segments, β_i_, will be given by the expression β_i_ = $\frac{\alpha_{\text{i}}}{E_{\text{i}}}$.

Figures 7E–G illustrates angular sector distribution options, displaying two dendrograms constructed using constant branch ending widths or constant neurite widths. From this figure, it can be seen that these two options favor either paying more attention to neurite distribution or to neurite *ending* distribution, depending on the ways α_i_ and β_i_ are chosen.

#### Ordering Criteria in Circular Dendrograms

2D microscopy images acquired from real neurons show neurites in fixed relative positions and orientations. However, their distribution is misleading; selecting another point of view or projection direction would normally result in different arrangements. By contrast, circular dendrograms replace projection-based distributions with user-defined synthetic arrangements. Using this synthetic approach, the way in which neurites are ranked can be modified in order to provide users with specific comparative information.

It is possible to modify angular positions at two levels: ordering of whole neurites, and ordering of individual branches. Nevertheless, it should be noted that the margin for designers to modify angular positions is notably smaller than for the cases of segment length and circular sector angle. Users might expect certain neurites or neurite fragments to be placed at a specific position. For example, users expect pyramidal neurons with the apical dendrite depicted departing vertically upward from the soma, with all of the segments belonging to the apical dendrite oriented in this direction. Also, they may expect axons departing downward from the soma. Introducing changes here might create confusion, although it is possible to devise solutions in which the orientation of certain principal neurites can be left unchanged, while other specific features might affect, for example, how basal dendrites are displayed.

**FIGURE 8:**
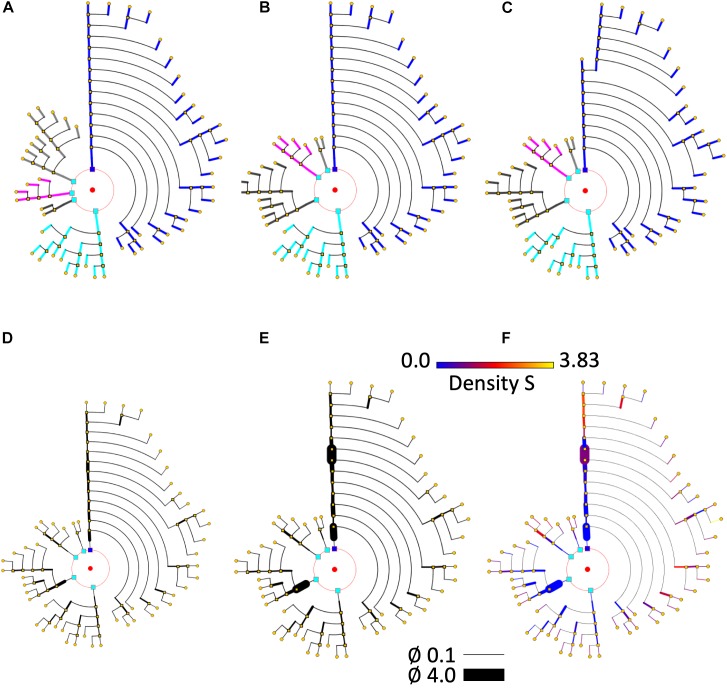
Different circular arrangements for the dendrites of the same neuron. **(A)** Original branch order. **(B)** Same neuron, with dendrites sorted according to arbor complexity (number of branch endings per dendrite). **(C)** Same neuron, with dendrites sorted according to their mean diameter. **(D)** Shows the pyramidal neuron dendrogram with segment widths proportional to the corresponding neurite segment widths. **(E)** Represents the same data, but using a gain factor to make width changes more evident, thereby facilitating user perception of relative segment width. **(F)** Color is used to display dendritic spine density. A pre-specified color map has been applied.

Many different ordering criteria can be selected for the circular arrangement of neurites and bifurcation nodes. Neurites could be ranked according to, for example, node count; bifurcation levels; total number of endings; number or density of dendritic spines; length, etc. With respect to criteria for the arrangement of the bifurcation nodes within neurites, features such as segment length, segment volume, and mean diameter can be used, among others. In fact, practically any feature can be used for the angular ordering of dendrites or branches, giving an indication of the distribution of that particular feature within a specific cell. Figures 8A–C illustrates the effect of using different arrangement criteria while displaying the same neuron.

### Mapping Additional Information Into Dendrograms

In addition to providing information about the basic structure of neurons, dendrograms can be used to present users with almost any kind of information acquired or calculated from raw data (regardless of whether the information is quantitative, ordinal, categorical, etc.). The data, obtained from the whole neuron or any of its parts, can then be mapped onto the dendrogram, combining in this way the symbolic representation with the real values of the extracted data. For this purpose, users have different options at their disposal. A number of visual resources have already been described in the previous sections. Some other resources, such as line width, color, etc., are described below.

#### Line Width and Color

The dendrogram segment width can convey information about the value of a specific variable on that neurite segment. If the dendrogram has been generated using real (or proportional) segment lengths, it provides a quite accurate idea of the changes of that variable along the neurites. Using uniform lengths, on the other hand, provides images that are generally easier to interpret.

Even though any variable can be mapped onto the dendrogram segment width, there are some features that are particularly suited to this kind of coding, such as dendrite width itself, dendrite spine density, average volume, etc., as illustrated by the neuron in Figures 8D–F.

Color has been frequently used in scientific visualization to facilitate the analysis of how specific parameters or variables change over a region of interest by mapping their intensity variations into colors through a modifiable color table or function. Also, using more than one color channel allows the display of more than one variable (Sheppard et al., 1969; Moreland and Moreland, 2009). Nevertheless, using color for information visualization requires some caution, and it is necessary to follow some basic design principles, since human perception of color presents here a number of problems and particularities (Rhyne, 2017). However, with an adequate design, coding information with color can provide useful feedback to users, particularly for numerical or categorical data. Figure 8F presents the same neuron as Figures 8D,E. In Figure 8F, color is used to also display dendritic spine density, by using a pre-specified color map.

#### Other Resources

Finally, there are other options for including additional information in dendrograms:

• Features associated with specific locations can be added to the dendrogram. If real lengths are used, the dendrogram reflects their distribution depending on soma distance. If unit segment lengths are used, feature variation depending on branching level is easier to perceive. An example of this is the placement of tags indicating the presence of dendritic spines or any other morphological feature.
• Textual tags can also be included to provide additional information, possibly at the request of the user.
• Pictograms and glyphs can also code feature information.
• Iteractive dendrogram modification provides users with a dynamic feedback that is very useful for enhancing user perception of certain aspects, such as —for example— how different features are distributed among neurites.

**FIGURE 9:**
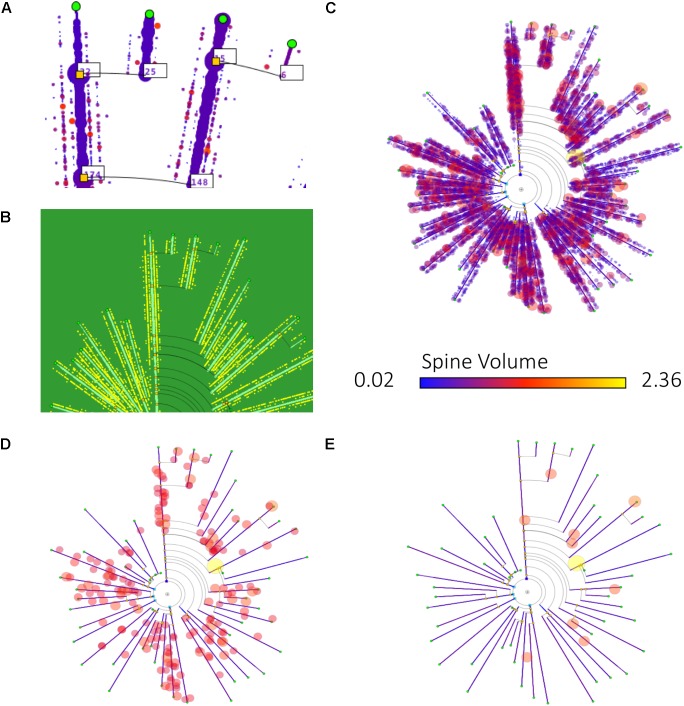
Different ways of mapping dendritic spine information onto dendrograms. **(A)** Includes the number of dendritic spines per branch in textual tags. **(B)** Dendritic spines are represented with equally sized dots on their corresponding dendrite positions. **(C–E)** Also display dendritic spines in their actual positions, represented as dots. In this last case, the dendrogram also displays dendritic spine volume information, by coding it as disk diameter and color, according to predefined scales. **(D,E)** Present the result of applying different filtering operations, with dendritic spines filtered out according to their volume (only those spines with volumes above a certain threshold are displayed).

Figure 9 shows some examples of how different information can be mapped onto dendrograms. In this case, real neurite lengths are shown (since the dendrograms are in RLM mode), and dendritic spine information has been added to the dendrogram segments, either as textual tags, dots or glyphs. (D) and (E) show the result of applying filtering operations to the spine data, displaying only those spines that have a volume above two different thresholds, so that only large spines are shown.

## Experimental Results

This paper describes a novel abstract representation model that can facilitate the visual exploration of neuronal morphologies. The proposed method can be applied to different tasks, from providing general overviews for characterizing a neuron or set of neurons, to the analysis of specific features of neuronal populations. Also, the proposed models are well suited to activities where users are interested in obtaining at a glance as much information as possible about neuronal morphology, such as when browsing through large neuronal repositories.

In general, the proposed method can be applied to data acquired by virtually any laboratory and technique. The dendrogram images included in this paper have been generated with HUGO (Highly Uniform Graphic Organization) — a software tool that can generate the models described.

Regarding the usefulness of the method, a preliminary validation study has been carried out with a set of users in order to evaluate the impact of visualizing symbolic dendrograms instead of conventional 2D projections of the 3D tracings, when performing analysis tasks related to the morphological structure of the cells. The results, presented below, show that using a symbolic representation enhances the perception of the underlying arborization structure while hiding finer, non-essential details and avoiding artifacts due to perspective projections.

Many different tasks can benefit from the kind of symbolic representations presented here. To illustrate this, we present various examples. First, a visual comparison of human and mouse neocortical pyramidal neurons. We then show the possibility of using HUGO for neuronal classification. Finally, we point out other domains where this model can be applied, such as for the analysis of brain vasculature.

### Analysis of Morphological Features: Counting the Number of Dendrites and Dendrite Bifurcation Nodes

A user study was conducted to evaluate how helpful the proposed symbolic representation is for tasks related to the analysis of some basic neuronal morphological features. Specifically, the experiment focused on counting the number of dendrites and the number of bifurcation nodes in a neuron.

#### Experimental Design

A set of seven neurons of different types was selected from the Neuromorpho repository (NeuroMorpho.Org, 2018). For each of them, two representations were computed — the first using the standard 2D projections of the neurons’ 3D morphological tracings, and the second using the circular dendrograms presented here. Seven images were rendered for each of the two representation methods. The selected neurons have different numbers of dendrites (between 4 and 8) and different numbers of bifurcation nodes (between 12 and 34). Table 1 shows the actual number of dendrites and nodes for each of the seven selected neurons.

**Table 1:** Number of dendrites and bifurcation nodes of the seven neurons involved in the validation experiment.

Neuron	N1	N2	N3	N4	N5	N6	N7
# Dendrites	4	5	5	5	6	7	8
# Nodes	13	12	13	19	26	21	34

Two different tasks were defined for this study: counting the number of dendrites and counting the number of bifurcation nodes for each of the neurons. Each participant was asked to perform both of these tasks by viewing the morphological tracings and the symbolic dendrograms of the cells, while the time required to complete both tasks with each depiction was measured. The dendrogram and 2D projection representations for all of the neurons were shuffled, in order to prevent participants from knowing beforehand the responses about node and dendrite count because the other representation of the same cell had just been analyzed.

#### Population and Procedure

To test the performance of the proposed symbolic representation system compared to the standard 3D representation, a group of 12 subjects counted the number of dendrites and nodes from all 7 neurons. This resulted in a total of 84 measurements of the number of dendrites, and 84 measurements of the number of nodes using the symbolic representation. The same tasks were performed using the standard 3D neuronal representations, resulting in a further 84 measurements of the number of dendrites and 84 measurements of the number of nodes.

Since the participants had no prior background in neuronal anatomy, a short explanation of the basic anatomical parts of the neuron was provided, together with a description of the symbolic model (using Figure 4 for this purpose). This explanation was the same and provided by the same person in all cases. Each user was then asked to count the number of dendrites and nodes for each image that was presented to him. As mentioned above, the images were selected in a random fashion, alternating between the symbolic and 3D depictions.

#### Results

The results of the tasks related to counting nodes and those corresponding to counting dendrites are shown in Table 2.

**Table 2:** Results of the node- and dendrite-counting task and time for completion of both tasks.

	Nodes				Dendrites				Time for completion of both tasks	
	*M*	*SD*	ARE	SDRE	*M*	*SD*	ARE	SDRE	AT	SDT
Real data	19.714	7.561			5.714	1.285				
3D experiment	14.309	6.057	0.279	0.141	6.273	1.434	0.160	0.157	26.428	11.756
Symbolic dendrogram	19.226	7.326	0.02	0.041	5.702	1.287	0.005	0.029	15.571	5.839

*M, mean; SD, standard deviation; ARE, average relative error; SDRE, standard deviation of the relative error; AT, average time; SDT, standard deviation time.*

The actual mean number of nodes per neuron for the experiment sample was 19.71, with a standard deviation of 7.56. The mean number of nodes counted by the subjects was 14.31 when using the standard 3D representations, and 19.23 when using the symbolic representations. The standard deviation of the observations when using the 3D representations was 6.06, while its value was 7.32 when using the symbolic representation (Table 2).

Regarding the average relative error, the observations performed using the 3D representations had a mean relative error of 0.28 and a standard deviation of 0.14. The relative error of the observations performed on the symbolic representations had a mean of 0.02, and a standard deviation of 0.04 (Table 2).

A similar trend can be seen when studying dendrites; the mean number of dendrites per neuron for the experiment sample was 5.71, with a standard deviation of 1.28. The mean number of dendrites counted by the subjects was 6.27 when the neurons were depicted using the standard 3D representations; and 5.70 when they were drawn using the symbolic representations. The standard deviation of the observations was 1.43 when the standard 3D representations were used; and 1.28 when the circular dendrogram representations were used (Table 2).

When the tests were carried out using the 3D representation, the mean relative error for the dendrite observations was 0.160, with a standard deviation of 0.157; while, with the symbolic representation, the mean relative error was 0.005, with a standard deviation of 0.029 (Table 2).

In both tasks (dendrite and node counting), the relative error obtained when using the symbolic model was one order of magnitude less than the error obtained while analyzing the 3D model.

The time taken by human operators to carry out the tasks was also markedly different, depending on the representation used. It took experimenters an average of 26.428 s to perform the morphologic analysis with the 3D representation (the standard deviation was 11.756 s). By contrast, it took only 15.571 s when using the symbolic model (the standard deviation was 5.839 s; Table 2). That is, using the proposed model, the time needed to perform the analysis was almost halved.

**FIGURE 10:**
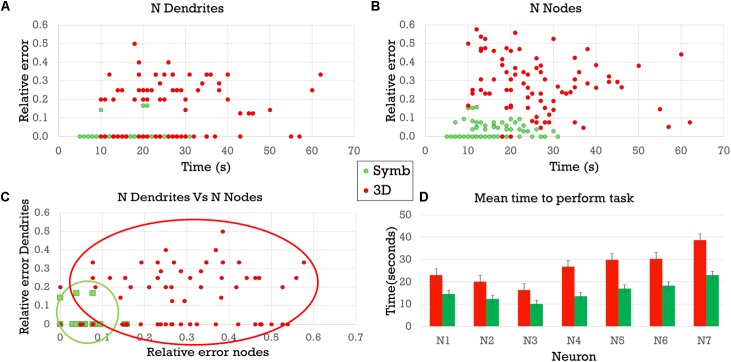
Relative errors and completion times for the two experimental tasks using the 3D representations and the symbolic dendrograms. **(A)** Relative errors when counting dendrites vs. completion time. **(B)** Relative errors when counting bifurcation nodes vs. completion time. **(C)** Relative errors when counting dendrites vs. relative errors when counting nodes. **(D)** Average completion time for performing the tasks.

These results are also shown in Figure 10 where (A) presents the relative error obtained when counting the number of dendrites, while (B) shows the relative error obtained when counting the number of nodes. In both panels, the horizontal axis shows the time taken to complete the visual analysis. It can be seen that the difference in relative errors is even greater when counting the bifurcation nodes, since this task is considerably more complex. (C) Depicts the errors of each task in each axis. Errors with the symbolic representation are confined to a small region in the proximity of the origin, while errors with the 3D representation present a remarkably higher variability. (D) Compares the completion time needed to perform both analysis tasks using each of the two representation models. The times were consistently lower when using the proposed circular dendrograms.

The numerical values previously presented suggest that the relative errors when counting dendrites and nodes are clearly lower when performing these tasks with the symbolic dendrogram. In order to statistically validate this affirmation, a *t*-test was performed stating the following null-hypothesis (*H*_0_): “There is no significant difference between the mean error when the task is performed using a 3D representation, and the mean error when the task is performed using the symbolic dendrogram.”

With regard to the dendrite counting task, the *t*-test analysis rejected *H*_0_ (*p*-value = 1.018E-15), and this was also the case for the node counting task in which the *t*-test analysis also rejected *H*_0_ (*p*-value = 3.188E-34).

In addition, the time needed to perform these analysis tasks was noticeably lower when using the symbolic dendrogram. A *t*-test validation, with the following null-hypothesis “There is no significant difference between the mean time to complete the tasks when they are performed using a 3D representation, and the mean time when they are performed using the symbolic dendrogram,” concluded that the differences in completion time are undoubtedly significant (*p*-value = 2,31499E-12).

These results point to a common conclusion: user performance when presented with the symbolic representations, both for counting nodes and dendrites, is remarkably better than when the observers are presented with morphologically accurate representations. The tests also seem to support the idea that the more complex the task, the larger the benefits of using simplified symbolic representations, as seen when comparing the dendrite counting task with the more complex node counting task.

Ultimately, these results prove that the symbolic model can be accurately and efficiently used to visually evaluate the morphological characteristics of neurons. However, the practical integration of this new symbolic model into current applications within the field of neuroscience would require an assessment of its performance when dealing with the different tasks that scientists face in their research, as well as a determination regarding which of the different representation variations described in this paper are better suited to different tasks.

### Browsing Through Large Neuronal Repositories

In addition to the visual analysis of numerical morphological variables (such as number of dendrites, number of nodes, etc.), other tasks can be carried out more easily when users have symbolic representations at their disposal. Some examples are given below.

**FIGURE 11:**
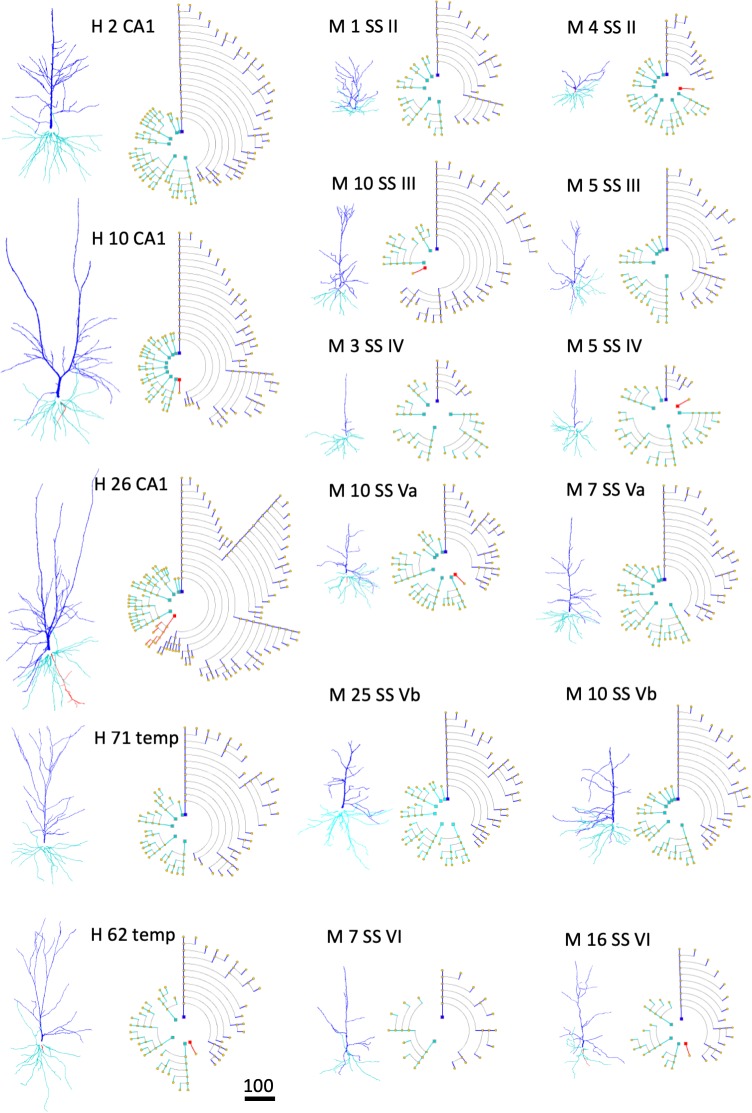
Examples of human (H) pyramidal neurons from CA1 of the hippocampus (CA1) and temporal cortex (temp), as well as from the mouse (M) somatosensory cortex (SS), through layers II, III, IV, Va, Vb, and VI. Each neuron is represented using both a 3D projection and a circular dendrogram. Note that the size of the cells are not represented in the models. Scale bar for the 3D projections = 100 microns.

An illustration of how symbolic representations facilitate the user experience is that the overall cell morphology can be more easily perceived with the help of circular dendrograms, especially when they are used in combination with 2D or 3D accurate representations. Neurons of different types —or those coming from different brain regions— present marked differences in their morphology. Dendrograms have the capacity to capture these anatomical differences and provide abstract representations that highlight such differences even at first glance. Figure 11 shows the dendrograms of apical and basal arbors from human pyramidal neurons obtained from human hippocampal field CA1 and temporal cortex, as well as from mouse somatosensory cortex (described in the section “Materials and Methods”). The dendrograms in this figure show clearly the huge visual variation among cells. Dendrograms, therefore, can also be useful when performing initial visual discriminations among cell populations.

As a second example, the complexity of neurite arbors is difficult to perceive when neurons are depicted in 3D, due to their intricate geometry and branch occlusion, as well as the fact that the image is dependent on the viewing angle; by contrast, symbolic dendrograms offer a clear, unambiguous representation of the cells’ morphology that aids the comparison of arborization patterns among sets of neurons.

Also, the simplicity of the symbolic representations can be beneficial when browsing through large repositories of neurons using circular dendrograms. In fact, the tests performed during the development of our tool allowed us to discover that one particular neuron had been included twice within a dataset of our laboratory — something that had remained unnoticed when exploring the dataset visualizing the 3D tracings in the usual way. Although this may seem anecdotal, it does further demonstrate the utility of the proposed representation model, since it allowed the users to flag up an issue that had gone unnoticed by a number of people.

**FIGURE 12:**
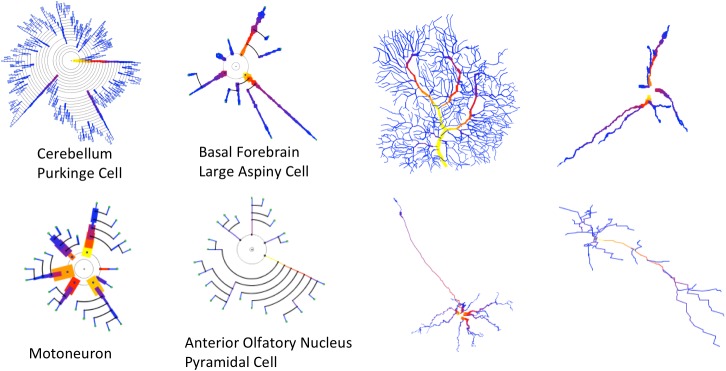
Four cells with very different morphologies, presenting their dendrograms **(left)** and morphological tracings **(right)**. The symbolic model preserves (and even emphasizes) morphological variations among cells. Line width and color provide increased information in the dendrograms. Purkinje Cell: Guinea pig, Cerebellum. NeuroMorpho.Org ID NMO_00610 (Rapp et al., 1994). Large Aspiny Cell: rat, ventral striatum. NeuroMorpho.Org ID NMO_04423 (McDonald et al., 2007). Motoneuron: rat, brainstem. NeuroMorpho.Org ID NMO_00665 (Nüñez-Abades et al., 1994). Anterior Olfactory Nucleus: rat, anterior olfactory nucleus. NeuroMorpho.Org ID NMO_05982 (Brunjes and Kenerson, 2009).

Finally, Figure 12 reinforces the conclusions drawn from the statistical analysis of the user experiment previously presented. It can be observed that counting the number of dendrites or bifurcation nodes can be performed much more easily by looking at the symbolic models. Using additional visual resources —such as color, to represent a specific variable (dendrite diameter in this example) — is also more effective in the symbolic representations, since the occlusions in the 3D representations hamper the perception of branch color.

Most of the examples presented so far have only dealt with arborization patterns. However, as discussed previously in this paper, the proposed method can make use of other resources, such as line width and color, to intensify certain morphological features. This can be useful for characterizing neuron families or for performing fast, approximated visual characterizations of neurons or neuron sets. In this regard, Figure 12 shows the morphologies of four very different kinds of neurons. The variability of their anatomy is effectively captured by the symbolic dendrograms, producing characteristic patterns that can be helpful for characterization or classification purposes.

### Application to Other Domains

The symbolic model presented in this paper has been specifically designed to represent neuronal morphology. Nevertheless, it can also be used in many other contexts.

Firstly, the representation of features other than morphological variables is straightforward. The visual resources described in this paper can be easily associated to variables of a very different nature, providing symbolic representations that can depict, for example, physiological features, morphological data, combinations of morphological and physiological data, etc.

**FIGURE 13:**
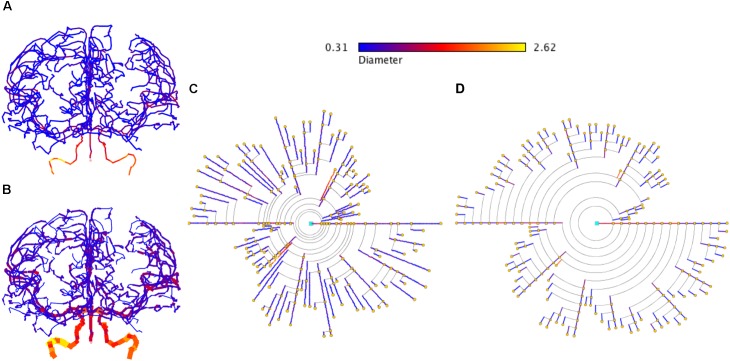
Representation of brain vasculature using circular dendrograms. **(A)** Shows the structure of brain vasculature, while **(B)** additionally depicts the diameter of the vessels. Circular dendrogram **(C)** corresponds to the image **(B)**, representing the diameter of the vessels according to the color scale shown in the same panel, using RLM for **(C)** and UM for **(D)**. SWC data model from http://cng.gmu.edu/brava: BG001 (Wright et al., 2013).

In addition, the proposed symbolic model can also be applied to other domains that present branching structures. Figure 13 shows an example of brain vasculature represented with circular dendrograms.

## Discussion, Conclusion, and Future Work

This paper describes a novel symbolic representation method for neurons, based on circular dendrograms. These diagrams encode user-selected features by varying visual characteristics or rearranging the different diagram elements according to predefined criteria. The flexibility provided by these changes facilitates the interactive parameterization of each visualization according to the users’ needs, emphasizing the most relevant variables while hiding the least important ones. By modifying their graphical characteristics, it is also possible to create sets of circular dendrograms that can give complementary views of the same data. These views can also be adapted to reflect the changing needs of users over time. It should be noted that the circular dendrograms described here can be automatically generated, providing deterministic models that are operator-independent.

This paper also presents other contributions:

• The idea that dendrograms can be tuned to enhance different kinds of information by customizing the way the information is encoded into the dendrogram, and specific proposals for modifying how this encoding is achieved.
• The proposal of using dendrograms that permit other information (morphological, functional, etc.) to be mapped onto them, such as the distribution of dendritic spines that is presented in this paper.
• The possibility of creating interactive and dynamic dendrograms. Exploring this option is beyond the scope of this paper, but will be done in a subsequent study.
• The idea of using dendrograms to perform specific analysis operations (i.e., Sholl analysis).

The concept of dendrograms is not new; classical, linear dendrograms have been in use for some time (Costa et al., 2000; Van Pelt et al., 2001; Cervantes et al., 2018), but the circular arrangement proposed in this work presents some advantages and interesting features:

• Clarity: the information displayed in circular dendrograms is easier to grasp, regardless of the level of user expertise. The system does not allow ambiguities that can mislead users, unlike the artifacts and variability inherent in 2D projections from 3D data. Compared with classical dendrograms, the new proposal provides representations that are closer to real cell morphologies.
• Simplicity: only essential, non-accessory information is included in the representations. Users can even collapse neurite portions for additional simplification, which also contributes toward improving the clarity of the representation.
• Precision: this new representation system allows the users to get an accurate idea of relative magnitude for ordinal or quantitative features, especially when additional graphical resources —such as color, width, angular spacing ordering, etc. — are correctly exploited. If the order and angular arrangement of dendrites follow the neuron’s 2D projections, circular dendrograms can also give a more precise indication of spatial distribution of features than classical dendrograms.
• Compactness: circular dendrograms make a better use of space than linear dendrograms.

Regarding clarity, circular dendrograms are an evolution of the traditional binary tree drawings of classical dendrograms. In addition to occupying less space, circular dendrograms, with their radial distribution of neurites, show a geometrical layout that is much closer to the real cell structure since neurites originate at and radiate from approximately central somas. It should be pointed out that one of the reasons why the use of traditional dendrograms has not become more widespread is possibly because they are not very natural, that is, their structure is very different from the real neuronal geometry.

Combining circular dendrograms with 2D projections can bring together the best of both worlds, showing the neurites’ real morphology while also displaying detailed but easier to analyze morphological information. Finding relationships among elements is easier with circular dendrograms than with 2D representations. Additionally, there are visual resources (such as distribution of angular width) that can be helpful for comparing different neurites and which are available only in circular dendrograms.

With respect to specific analysis tasks, the results presented in Section “Experimental Results” support the considerations stated above, showing that the representation model presented in this paper can help to perform visual exploratory analysis tasks, such as the analysis of morphological variables, discrimination among neurons, and the exploration of global features of neuronal morphology. Additionally, we believe that this kind of representation will be very useful for browsing large neuronal repositories, very possibly in combination with 2D projections of the real neuronal morphologies. A study comparing different populations (e.g., experts vs. people with no prior exposure to neuroscience) would be also of great interest.

There is a wide scope for future studies, focusing on topics such as the integration of circular dendrograms in interactive exploratory analysis tools; the inclusion of other morphological or electrophysiological variables and connectivity information; exploitation of the temporal dimension in dynamic dendrograms; and the enhancement of the capabilities of the method regarding multiple levels of detail in complex scenarios. In addition, in-depth user studies should be carried out in close collaboration with neuroscience laboratories to fully assess the potential of the method.

One final point that should be made is that the method presented here approaches just one of the multiple aspects involved in the study of neuroanatomy, but it approaches it in a novel way, that is, through the design of a formal representation system. This representation system can be used either with experimentally acquired data or with synthetic data, derived from and possibly validated by *in silico* simulations. We believe that it is a small but important step in a direction that could greatly affect scientific workflows through the introduction of computer supported-analysis tools.

## Author Contributions

JJA designed and developed the methods, did the image processing and did software development. SM and LP supervised the work and discussed the method’s design and the results. JJA, SM, LP, JD, and RB-P wrote the paper.

## Conflict of Interest Statement

The authors declare that the research was conducted in the absence of any commercial or financial relationships that could be construed as a potential conflict of interest.
